# Photo-Fenton and TiO_2_ Photocatalytic Inactivation of Model Microorganisms under UV-A; Comparative Efficacy and Optimization

**DOI:** 10.3390/molecules28031199

**Published:** 2023-01-26

**Authors:** Eirini Kanata, Ioannis Paspaltsis, Sotiris Sotiriadis, Chrysanthi Berberidou, Sophia Tsoumachidou, Dimitra Dafou, Konstantinos Xanthopoulos, Minas Arsenakis, Athanasios Arsenakis, Ioannis Poulios, Theodoros Sklaviadis

**Affiliations:** 1 Laboratory of Pharmacology, Department of Pharmacy, School of Health Sciences, Aristotle University of Thessaloniki, 54124 Thessaloniki, Greece; 2 Department of Genetics, Development and Molecular Biology, School of Biology, Aristotle University of Thessaloniki, 54124 Thessaloniki, Greece; 3 Laboratory of Physical Chemistry, Department of Chemistry, Aristotle University of Thessaloniki, 54124 Thessaloniki, Greece; 4 Centre for Research and Technology Hellas, Institute of Applied Biosciences, 57001 Thermi, Greece; 5 Laboratory of General Microbiology, Department of Genetics Development and Molecular Biology, School of Biology, Aristotle University of Thessaloniki, 54124 Thessaloniki, Greece; 6 STERIMED SA, G’ Fassi, Building Block 52b, Sindos Industrial Area, 57022 Thessaloniki, Greece

**Keywords:** photocatalytic oxidation, TiO_2_, photo-Fenton, microorganism inactivation, *Bacillus stearothermophilus* spores, MS2, *Staphylococcus aureus*, Escherichia coli

## Abstract

Photocatalytic inactivation of pathogens in aqueous waste is gaining increasing attention. Several homogeneous and heterogeneous photocatalytic protocols exist using the Fenton’s reagent and TiO_2_, respectively. A comprehensive study of homogeneous and heterogeneous photocatalysis on a range of microorganisms will significantly establish the most efficient method. Here, we report a comparative study of TiO_2_- and Fe^+3^-based photocatalytic inactivation under UV-A of diverse microorganisms, including Gram-positive (*Staphylococcus aureus*) and Gram-negative (*Escherichia coli*) bacteria, bacterial spores (*Bacillus stearothermophilus* spores) and viruses (MS2). We also present data on the optimization of TiO_2_ photocatalysis, including optimal catalyst concentration and H_2_O_2_ supplementation. Our results indicate that both photo-Fenton and TiO_2_ could be successfully applied for the management of microbial loads in liquids. Efficient microorganism inactivation is achieved with homogeneous photocatalysis (7 mg/L Fe^+3^, 100 mg/L H_2_O_2_, UV-A) in a shorter processing time compared to heterogeneous photocatalysis (0.5 g/L TiO_2_, UV-A), whereas similar or shorter processing is required when heterogenous photocatalysis is performed using microorganism-specific optimized TiO_2_ concentrations and H_2_O_2_ supplementation (100 mg/L); higher H_2_O_2_ concentrations further enhance the heterogenous photocatalytic inactivation efficiency. Our study provides a template protocol for the design and further application for large-scale photocatalytic approaches to inactivate pathogens in liquid biomedical waste.

## 1. Introduction

Photocatalytic oxidation has come to the fore as an alternative and environmentally friendly method for inactivation of hazardous substances and microorganisms in aqueous solutions, with extensions to the treatment of drinking water [1,2], urban and industrial wastewater [3,4,5,6,7,8,9] and more recently to biomedical liquid waste [10,11].

Photocatalytic oxidation, as an advanced oxidation process (AOP), is based on the production of reactive oxygen species (ROS) or free radicals such as superoxide radicals (O_2_**^−^**), hydroperoxyl radicals (HO_2_**^.^**) and hydroxyl radicals (HO˙), which non-selectively attack organic molecules, eventually resulting in the formation of CO_2_ and inorganic salts [2]. Several photocatalytic approaches have been reported, using catalysts either occurring in the same phase as the reactants (homogeneous) or not (heterogeneous)**.** The use of Fenton’s reagent (Fe^+2^ and H_2_O_2_) and TiO_2_ in the context of homogeneous and heterogeneous photocatalytic oxidation, respectively, has been extensively studied [12,13,14,15]. In the photo-Fenton process, HO˙ and O_2_^.−^ are generated during the irradiation of the H_2_O_2_ and Fe^2+^ mixture (Fenton’s reagent) in acid conditions. The addition of oxalic acid to the solution containing Fe^+3^ leads to the formation of ferrioxalate complexes (ferrioxalate-assisted photo-Fenton process) that under irradiation can also produce oxidative species such as O_2_^.−^, HO_2_˙ and HO˙ radicals. TiO_2_-based photocatalysis is initiated with the irradiation of TiO_2_ with a photon of energy equal to or greater than its band gap width, and the formation of photogenerated electron/hole (e−/h+) pairs. In aqueous suspensions the produced holes (h + VB) and electrons (e−CB), can react with surface HO^−^ groups and O_2_, respectively, leading to the formation of HO˙ and O_2_^.−^ radicals [8].

Extensive research has been conducted aiming at the development of optimized homogeneous and heterogeneous photocatalytic approaches for the decomposition and detoxification of hazardous organic substances in aqueous solutions, such as antibiotics [13,15,16,17], pesticides [18,19,20] and dyes [21,22,23,24]. Similar studies have been conducted for the evaluation of the photocatalytic inactivation of microorganisms in suspension [25], focusing on endospores, considered to be one of the most resistant targets [12,26,27], viruses [28,29,30], fungi [14], Gram-positive and Gram-negative bacteria [31].

Microorganism inactivation mediated by photo-Fenton and TiO_2_ photocatalysis is achieved through different pathways depending on the microbe. In general, microorganisms’ photocatalytic inactivation represents the synergistic effect of UV light and oxidative radicals generated by TiO_2_ or Fe^+3^ following UV irradiation. UV-A affects microbial proteins and nucleic acids through ROS production. Previous studies aiming to delineate the mechanism of TiO_2_-based photocatalytic inactivation reported catalyst adsorption on the microbes’ surface. Next, the ROS react with organic molecules on the microbes’ outer surface, such as proteins (porins, proteins involved in oxidative stress response, in transport, and in bacterial metabolism) and polyunsaturated fatty acids [32], and destroy the cell wall and membrane, as evidenced by scanning electron microscopy [12,33]. Interestingly, it has recently been reported that different TiO_2_ configurations generate different types of ROS, exerting differential bactericidal efficacies [34]. In the case of viruses, ROS attack the phospholipid bilayer and the envelope and/or capsid proteins [35]. In all cases, damage of the microbes’ outer surface allows ROS penetration inside the cell and leakage of cellular contents [33]. Having gained access to the cytoplasm, free radicals promote oxidative stress, attack and decompose cellular enzymes. Oxidative stress induces antioxidant cellular responses to counter the effects; however, enzymes mediating such responses are affected by oxidative agents, resulting in malfunction of antioxidant defense. Moreover, oxidative species attack and destroy nucleic acids, further contributing to microorganism inactivation. Similar effects have been reported in photo-Fenton approaches. Regarding microorganism-specific inactivation mechanisms, in the case of MS2, photo-Fenton inactivation is mainly exerted through oxidant generation in the bulk, in contrast to bacterial inactivation, which is mostly attributed to intracellular oxidative-stress [36].

Previous studies reported that *B. stearothermophilus* spores are more efficiently inactivated using 0.1 g/L TiO_2_, resulting in complete inactivation following 90 min treatment [12]. Optimal TiO_2_ concentration for *S. aureus* inactivation has been reported to be 0.1 g/L [31]; for MS2 inactivation, previous studies used 1 g/L TiO_2_, and required irradiation (300 to 420 nm) treatment for 120 min to achieve 0.95-log inactivation [37]. Several studies have been conducted on TiO_2_-based *E. coli* inactivation (some of them reviewed in Foster et al. 2011 [33], among other microorganisms). In a study by Khani et al. in 2016, following catalyst concentration titration experiments, 0.5 g/L was determined as the optimal TiO_2_ concentration for *E. coli* inactivation under irradiation of 300–420 nm, after a processing time of 150 min [38]. Regarding photo-Fenton approaches, a 9-log inactivation of *Bacillus subtilis* spores was achieved under UV-A irradiation at 365 nm using 2.5 mM Fe^+2^ and 100 mM H_2_O_2_ [26]. Exposure to 600 W/m^2^ irradiation for 30 min in the presence of Fe^+3^ and H_2_O_2_ at a 1:1 ratio resulted in a 4-log MS2 reduction [39].

Most of the previous studies focused either on the homogeneous or the heterogeneous inactivation of individual microorganisms, and only few addressed the comparative effectiveness of photocatalytic inactivation on different microorganisms [37,40,41,42]. Currently, there is no study addressing the efficiency of UV-A mediated homogeneous and heterogeneous photocatalysis against different microorganisms under the same experimental set-up. Data generated under different experimental conditions may only provide indirect insights into the effectiveness of photocatalytic oxidation approaches on microorganisms’ inactivation.

Here, we report the comparative effectiveness of TiO_2_- (heterogeneous) and Fe^+3^- (photo-Fenton, homogeneous) based photocatalysis under UV-A irradiation against a range of microorganisms including bacterial spores (*Bacillus stearothermophilus* spores) as a representative of the most resistant to inactivation targets, MS2 bacteriophage as a model of human enteroviruses, *Staphylococcus aureus* and *Escherichia coli* as representatives of Gram-positive and Gram-negative bacteria, respectively, at concentrations similar to pathogen titers in biological fluids of patients with bloodstream infections.

We also present data on the optimization of heterogeneous photocatalytic approaches, entailing catalyst concentration and H_2_O_2_ supplementation. Our data indicates that both photo-Fenton and TiO_2_ could be successfully applied for microbe inactivation. Indeed, efficient microorganism inactivation is achieved with homogeneous photocatalysis (7 mg/L Fe^+3^, 100 mg/L H_2_O_2_, UV-A) in a shorter processing time compared to heterogeneous photocatalysis (0.5 g/L TiO_2_, UV-A), whereas similar or shorter processing is required when heterogenous photocatalysis is performed under microorganism-specific optimized TiO_2_ concentrations and H_2_O_2_ supplementation (100 mg/L or 1000 mg/L). 

## 2. Results

### 2.1. Comparative Assessment of Homogeneous and Heterogeneous Photocatalysis for the Inactivation of Model Microorganisms

We report the effectiveness of TiO_2_- and photo-Fenton-based photocatalytic oxidation on the inactivation of microorganisms representative of different microbial groups. These groups of microorganisms are detected in biomedical liquid waste [43] and display different degrees of resistance against conventional inactivation methods, such as chemical treatment (chlorine or ozone) [44] irradiation (UV) [45,46], electro-thermal-deactivation (ETD) [47] and microwaving [48].

To assess microorganism inactivation, we estimated viability reduction (Nt/N0) and inactivation efficiency (I) following treatment with TiO_2_ (0.5 g/L) or with the photo-Fenton reagent (7 mg/L Fe^+3^, 100 mg/L H_2_O_2_) under UV-A irradiation (Figure S1A,B, Supplementary Materials). We observed a shorter processing time required for microorganism inactivation under homogeneous photocatalysis; indeed, homogenous photocatalysis achieved a 3-log reduction of *B. stearothermophilus* spores within 60 min of processing and resulted in no bacterial growth after 180 min of treatment. In contrast, the heterogeneous approach achieved a 3-log reduction at 300 min of treatment. Similarly, for MS2 inactivation, no plaque formation was detected in samples collected after 30 min of processing with TiO_2_, while the same result was achieved following a 5 min treatment with the photo-Fenton reagent. Intriguingly, *S. aureus* presented a distinct pattern of inactivation, according to which both homogeneous and heterogeneous approaches resulted in no bacterial growth following 30 min of treatment; however, intermediate timepoints suggest a sharper decline of *S. aureus* viability through heterogeneous photocatalytic oxidation. Based on the existing literature, we speculate that this observation may be associated with: (a) the tendency of *S. aureus* to aggregate at high concentrations [49,50], rendering it less accessible to the chemical attack by the produced oxidizing agents, and (b) with the absorbance of TiO_2_ particles on the microorganisms’ surface [51], providing a direct contact between the pathogen and oxidizing agents. *E. coli* inactivation was only assessed in the case of heterogeneous photocatalysis, since it is highly susceptible to the low pH required for the photo-Fenton reagent. *E. coli* was the most susceptible among the tested microorganisms, resulting in no growth following 5 min of treatment with TiO_2_.

### 2.2. Heterogeneous Photocatalysis Optimization

We next aimed to improve the performance of heterogeneous photocatalytic oxidation. To this end, we tested different catalyst concentrations (0.1 g/L, 0.5 g/L, 1 g/L) and further supplemented the best performing condition with increasing concentrations of H_2_O_2_ (100 mg/L, 500 mg/L, 1000 mg/L). Table 1 summarizes heterogeneous photocatalysis optimization results. We noticed that the lower catalyst concentrations (0.1 g/L, 0.5 g/L) were more effective in most of the cases; in particular, the lowest catalyst concentration (0.1 g/L) resulted in optimal inactivation in the cases of *B. stearothermophilus spores, S. aureus* and MS2; intermediate catalyst concentration (0.5 g/L) performed equally well in the cases of *S. aureus* and MS2, while it was most efficient in *E. coli* inactivation. These observations are in line with previous studies suggesting that higher TiO_2_ concentrations may promote nanoparticle aggregation, resulting in light scattering and thus reduced efficiency [52]. We also noticed that increasing H_2_O_2_ concentrations significantly enhance inactivation efficiency. This effect should be attributed to increased hydroxyl radical production, due to H_2_O_2_ photolysis. Under optimal heterogeneous photocatalysis, complete inactivation of *B. stearothermophilus* spores was achieved following 60 min treatment (0.1 g/L TiO_2_, 1000 mg/L H_2_O_2_); *S. aureus* and MS2 were completely inactivated following 5 min treatment at medium TiO_2_ (0.5 g/L) and medium to high H_2_O_2_ (500 mg/L, 1000 mg/L) concentration, whereas treatment for only 2 min was sufficient to inactivate *E. coli* at 0.5 g/L TiO_2_ supplemented with low H_2_O_2_ concentration (100 mg/L).

### 2.3. Comparative Assessment of Homogeneous and Optimized Heteogeneous Photocatalysis for the Inactivation of Model Microorganisms

**Figure 1:**
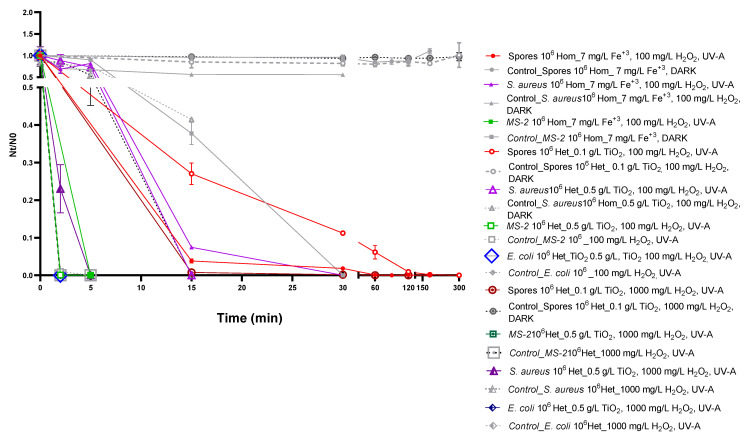
Homogeneous (7 mg/L Fe^3+^, 100 mg/L H_2_O_2_, pH 3.0, UV-A, solid symbols) and heterogeneous photocatalysis optimized for microorganism-dependent TiO_2_ concentration (no fill symbols) supplemented with either 100 mg/L H_2_O_2_ (continuous lines) or 1000 mg/L H_2_O_2_ (dashed lines) for the inactivation of 10^6^ cfu/mL *Bacillus stearothermophilus* spores (red), 10^6^ pfu/mL MS2 (green), 10^6^ cfu/mL *Staphylococcus aureus* (purple) and 10^6^ cfu/mL *Escherichia coli* (blue). The graph depicts viability reduction (Nt/N0) of treated samples and corresponding controls (grey symbols and lines) in relation to processing time. Zero values in the Y axis represent cases for which no microorganism growth was detected (0 cfu/mL or pfu/mL). Error bars correspond to standard errors from triplicates. The legend shows experimental conditions for processed samples. *Escherichia coli* is highly susceptible at pH 3 required for homogeneous photocatalysis, and thus not included in these experiments.

Next, we compared the efficacy of homogeneous and optimized heterogeneous photocatalysis (Figure 1 and Figure S2). To this end, we plotted viability reduction (Nt/N0) as determined for the homogeneous (7 mg/L Fe^3+^, 100 mg/L H_2_O_2_, pH 3.0, UV-A) and heterogeneous approach. Heterogeneous photocatalysis was performed under microorganism-specific TiO_2_ concentration optimized conditions, in the presence of 100 mg/L H_2_O_2_, similar to the H_2_O_2_ concentration used for homogeneous photocatalysis. In addition, data obtained from heterogeneous photocatalysis performed under the before-mentioned optimized conditions, but in the presence of the highest H_2_O_2_ concentration tested (1000 mg/L) were also plotted to visualize the effect of increasing H_2_O_2_ concentrations on inactivation efficiency (Figure 1 and Figure S2).

Our data shows that under the same H_2_O_2_ concentration (100 mg/L), both approaches require a processing time of 180 min for *B. stearothermophilus* spores’ inactivation. Similarly, for MS2, the optimized heterogeneous photocatalysis performed equally well compared to the homogeneous approach, allowing complete inactivation after 5 min of treatment. On the other hand, photocatalytic inactivation of *S. aureus* was achieved within a 2-fold shorter processing time (15 min) under the optimized heterogeneous approach in the presence of 100 mg/L H_2_O_2_, highlighting the microorganism-specific differences of the tested approaches in terms of efficiency.

Interestingly, further reduction of the required processing time was observed, when heterogeneous photocatalysis was performed using higher H_2_O_2_ concentration (1000 mg/L). Indeed, under these conditions, a 3-fold reduction of the processing time for inactivation of *B. stearothermophilus* spores was observed (from 180 min for homogeneous to 60 min for optimized heterogeneous photocatalysis). Similarly, a reduction of processing time by a factor of 6 (from 30 min for homogeneous to 5 min for optimized heterogeneous photocatalysis) was observed for the inactivation of *S. aureus*; in the case of MS2, higher H_2_O_2_ supplementation resulted in complete inactivation after 5 min of treatment. Similarly, H_2_O_2_ supplementation in heterogeneous photocatalysis further reduced the processing time required for *E. coli* inactivation to 2 min compared to 5 min for the originally tested, non-optimized conditions.

A detailed optimization of homogeneous photocatalysis was not considered in this study, due to the initial observation that homogeneous outperforms heterogenous photocatalysis. However, we did assess the effects of different Fe^+3^ concentrations (7 mg/L, 14 mg/L) on the inactivation of the most resistant microorganisms, namely *B. stearothermophilus* spores and *S. aureus*. Our data indicate that the Fe^+3^ concentrations tested deliver similar results. In addition, we assessed the effect of increasing H_2_O_2_ concentration (100 mg/L, 500 mg/L, 1000 mg/L) on *S. aureus* inactivation, verifying that, similar to heterogeneous photocatalysis, supplementation with increasing H_2_O_2_ concentration improves inactivation efficiency (data not shown). Interestingly, optimized homogeneous conditions achieved complete inactivation of *S. aureus* following 5 min of treatment, similar to the optimized heterogeneous photocatalysis (Figure 1, Table 1), further supporting that both photocatalysis approaches are efficient for microorganism inactivation.

## 3. Discussion

We have conducted a comparative study to assess the effectiveness of photo-Fenton- and TiO_2_-based photocatalysis under UV-A irradiation on the inactivation of a range of microorganisms in suspension, in a laboratory scale. Several studies on microorganism inactivation through photocatalytic oxidation approaches have been reported (reviewed by Venkata Laxma Reddy et al. 2017 [53] and by Bono et al. 2021 [35] for TiO_2_-based methods). Heterogeneity in terms of experimental procedures such as catalyst configuration and concentration, irradiation source (e.g., artificial, solar), irradiation wavelength and intensity, photocatalytic set-up configuration, operational pH (especially for Fenton and Fenton-like approaches) and microorganism strains, as well as their initial concentrations tested, hampers a direct comparison of the published results. Even in studies where most of the tested conditions were similar (e.g., catalyst configuration and concentration, microbial strains and concentration), variation associated with the utilized irradiation does not allow direct comparison of inactivation efficiency among previous studies or with the present study. We aimed to comparatively assess the effectiveness of different photocatalytic approaches on different microorganisms’ inactivation. This necessitates testing under the same experimental set-up, not previously conducted for TiO_2_- and Fenton-based approaches against the microorganisms tested in this study. Our study has been performed in a laboratory scale using individual microorganisms and is, to our knowledge, the first study that comparatively assesses the before-mentioned photocatalytic approaches on the inactivation of the studied microorganisms under the same experimental conditions.

We tested a range of model microorganisms, representative of different microbial groups (bacterial spores—*Bacillus stearothermophilus spores*, viruses—MS2, as a surrogate of human enteroviruses, Gram-positive—*Staphylococcus aureus* and Gram-negative -*Escherichia col*i, bacteria). Differential resistance to inactivation following photocatalytic oxidation was observed, displaying the same pattern irrespectively of the photocatalytic method utilized. The following classification in descending order was detected; *B. stearothermophilus spores* > *S. aureus* > MS2 > *E. coli*. This classification is in tandem with previous studies comparing the efficiency of microorganisms’ photocatalytic inactivation under the same experimental conditions, highlighting the high resistance of bacterial spores [54]. Similarly, MS2 has been reported as more resitant to photocatalytic inactivation compared to *E. coli* [37,55,56], whereas contradictory results have been reported on the resistance of Gram-positive (*S. aureus*) and Gram-negative (*E. coli*) bacteria. In particular, and in line with our data, the Gram-positive *S. aureus* has been reported to be more resistant compared to the Gram-negative *E. coli* [57,58,59,60], in contrast to other studies reporting the opposite [40,41,61].

The observed differential resistance to photocatalytic inactivation may be related to the size, structure and chemical composition of the tested microorganisms. Previous studies focusing on TiO_2_-based photocatalytic inactivation, reported that the catalyst is absorbed on the surface of the microbes. Produced reactive oxygen species attack and destroy the microbes’ outer surface [12,33], allowing ROS penetration inside the cell and leakage of cellular contents, followed by cell lysis and complete mineralization of the microorganism [33]. Structural elements affect the rate of oxidative attack. In this regard, spores, consisting of a thick, multilayer coat, are expected to be more resistant to photocatalytic inactivation [33]. Similarly, differences in the cell wall structure between Gram-positive and Gram-negative bacteria may contribute to their differential susceptibility to photocatalytic inactivation. In addition, species-specific differences related to oxidative stress responses, such as production of catalase by *S. aureus*, may contribute to the observed differential susceptibilities to photocatalytic oxidation. In addition, differences related to nucleic acid repair mechanisms, which are lacking in MS2, may explain this microorganism’s high susceptibility, possibly associated with higher vulnerability to nucleic acid damage through ROS [62].

Our data highlights efficient microorganism inactivation with both photo-Fenton and TiO_2_ in a relatively short processing time. Efficient microorganism inactivation is achieved with homogeneous photocatalysis (7 mg/L Fe^+3^, 100 mg/L H_2_O_2_, UV-A) in a shorter processing time compared to the heterogeneous approach (0.5 g/L TiO_2_, UV-A). Similar or shorter processing time is required when heterogenous photocatalysis is performed under microorganism-specific optimized TiO_2_ concentrations and H_2_O_2_ supplementation (100 mg/L or 1000 mg/L). Even though a detailed optimization of homogeneous photocatalysis conditions was not within the scope of this study, we did observe enhanced inactivation efficiency when homogeneous photocatalysis was performed using higher H_2_O_2_ concentrations.

Collectively, our data indicate that both homogeneous and heterogeneous photocatalysis are efficient for microorganism inactivation in suspension; following optimization and H_2_O_2_ supplementation, heterogeneous photocatalysis performance is significantly improved, resulting in a similar or better inactivation efficiency compared to the homogeneous approach. In addition, further improvement of homogeneous photocatalysis efficiency may be achieved through additional H_2_O_2_ supplementation, in an H_2_O_2_ concentration-dependent manner, as indicated by our experimentation on *S. aureus*.

Thus, both photo-Fenton and TiO_2_ can be successfully applied for the management of microbial loads in liquids. Consideration of additional approach-specific aspects would further enable the selection of the most suitable method. These include the low toxicity and negligible biological effects of TiO_2_, its efficient photoactivity, resistance against photocorrosion (high chemical stability) and acids [2,63], and the possibility to reuse this catalyst, reducing the total operational costs. Moreover, optimization of the TiO_2_ efficiency may be achieved in a cost-efficient way by supplementation with H_2_O_2_. On the other hand, the photo-Fenton approach requires a much cheaper catalyst and delivers efficient inactivation within relatively low treatment time; however, this approach results in the production of sludge, making difficult the isolation and re-use of the catalyst. In addition, operational costs associated with pH adjustment and sludge removal before final disposal should be also considered [64]. As an alternative, the application of a hybrid photocatalytic model has been proposed and reported to be up to 1.5 times more efficient than the individual processes [65].

This work is considered a template for follow-up studies on large scale liquid biomedical waste for identification of the best performing conditions. Biomedical waste produced by hospitals and biochemical laboratories harbor hazardous, toxic substances and pathogenic microorganisms and require efficient inactivation prior to final disposal. Efficient biomedical waste management is necessary in terms of protecting the environment and ensuring public health, and currently represents one of the biggest challenges. The application of advanced oxidation processes for the inactivation of hazardous chemicals [11] and microorganisms [66] in medical liquid waste is emerging as a promising, efficient, sustainable and environmentally friendly approach.

## 4. Materials and Methods

### 4.1. Microorganisms and Culture Conditions

All experiments were conducted using model microorganisms; *Bacillus stearothermophilus* (ATCC 7953, spores), MS2 bacteriophage (ATCC 15597-Β1), *Staphylococcus aureus* (ATCC 6538, Gram-positive bacteria) (kindly provided by Emeritus Professor Minas Arsenakis, Laboratory of General Microbiology, Department of Genetics Development and Molecular Biology, School of Biology, Aristotle University of Thessaloniki, Greece, 54124) and *Escherichia coli* (XL1-blue, Gram-negative bacteria). MS2 presents similarities in terms of size, shape and genetic material type (RNA) with human enteroviruses, and thus was included in our study as a non-pathogenic simulator of these pathogens.

Microorganisms were propagated in Tryptic Soy Broth/Agar-TSB (casein peptone 17 g/ L, soya peptone 3 g/ L, K_2_HPO_4_ 2.5 g/ L, NaCl 5 g/ L, glucose 2.5 g/L, for *Bacillus stearothermophilus*) and Luria-Bertani-LB (10 g/L tryptone, 5 g/L yeast extract and 5 g/L NaCl, for *S. aureus and E.coli*). In the case of *E. coli*, LB was supplemented with 12 μg/mL tetracycline (LB-tet). LB was used for propagation of the MS2 host *E. coli* Top10F’ strain. Agar plates of the before mentioned media, containing 16 g/L of bacteriological agar, were used for microorganisms’ quantification. The top agar method was applied in order to assure equal distribution of the plated microbes. eTop agar of each media, contained 7 g/L of agar. 

*Bacillus stearothermophilus* spores were initially purchased, impregnated on paper strips (ATCC 7953, 10^6^ cfu/strip) and processed as previously described [12] for the preparation of endospore working stocks. MS2 stocks containing 2 × 10^11^ plaque forming units (pfu) were used for the preparation of MS2 working stocks. MS2 was propagated through infection of an early log phase *E. coli* Top10F’ liquid culture in LB-tet. To this end, 100 mL of LB-tet media were inoculated with a single *E. coli* Top10F’ colony from a fresh LB-tet agar Petri dish. The culture was incubated at 37 °C under constant agitation, until OD_600_ reached a value of 0.15. Simultaneously, 2 μL of the phage stock were added. The culture was left at rest for 5 min in the incubator to allow bacterial infection and was then incubated for 20 h at 37 °C, under constant agitation. After centrifugation (20 min, 3.500× *g* at 4 °C), the phage-containing supernatant was filtered through a 0.2 nm syringe filter and stored at 4 °C. Typical yields of the procedure ranged between 2 to 5 × 10^11^ pfu mL^−1^. The exact titer of the *B. stearothermophilus* and MS2 preparations was determined by colony counting of plated serial dilutions. To this end, *B. stearothermophilus* spores were activated by boiling for 5 min before plating, while MS2 samples were incubated with a fresh host *E. coli* Top10F’ culture before plating. Stock *E. coli and S. aureus* vials containing 1 mL of corresponding o/n cultures supplemented with 10% glycerol were used for setting 50 mL liquid cultures.

### 4.2. Chemical Reagents

Heterogeneous photocatalysis was conducted with TiO_2_ P25 (Degussa, anatase/rutile = 7/3, S_BET_ (Brunauer–Emmett–Teller specific surface area) = 55 ± 15 m^2^/g, particle size/diameter = 21 nm, CAS No 13463-67-7). For the homogeneous photocatalysis process, the photo-Fenton (Fe^3+^/H_2_O_2_/UV-A) reagent (FeCl_3_, Chem-Lab, Cat No CL00.0910.1000) was used in acid conditions (pH 3) [12]. All other culture media and chemical reagents used for microorganism growth and buffer preparation were purchased from Applichem.

### 4.3. Photocatalytic Inactivation

**Figure 2:**
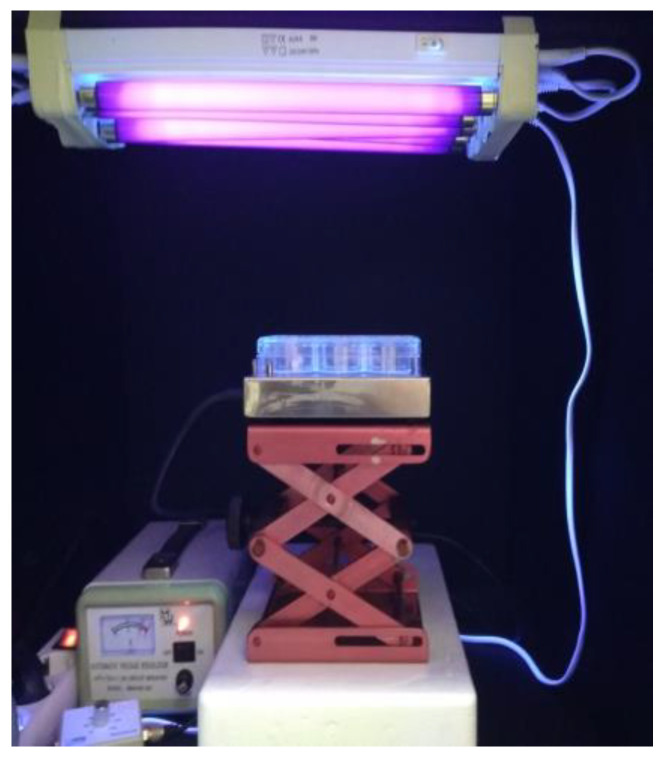
Configuration of the photocatalytic set-up. The photograph depicts the bench-scale photocatalytic reactor used in this study. UV-A lamps connected to a voltage stabilizer and emitting at a peak of 365 nm were used as a source of irradiation. Photocatalysis was performed in 6-well plates placed at a distance of 10 cm from the light source. A magnetic stirrer was used to ensure constant stirring of the treated samples.

Photocatalytic oxidation of microorganisms was performed in a bench-scale photocatalytic reactor (Figure 2), equipped with five parallel UV-A lamps as a light source (TLD 8 W/08, Phillips, emitting light with a spectral peak centered on 365 nm, 30 cm long, connected to a voltage stabilizer). Experiments were performed in 6-well plates under constant stirring (400 rpm) by a magnetic stirrer, in a final volume of 10 mL. The reaction plates were placed at 10 cm from the irradiation source. The intensity of the incident irradiation received by the treated samples at this distance was measured using a Photometer/Radiometer PMA 2100 (Solar Light Co., Glenside, PY, USA) equipped with a UV-A detector and determined to be 4.59 mW cm^−2^. Microorganisms were added at a final concentration of 10^6^ colony forming units per milliliter (cfu/mL) or 10^6^ plaque forming units/milliliter (pfu/mL), similar to the levels of pathogen titers in biological fluids of patients with bloodstream infections, representing the highest possible concentrations in biomedical liquid waste [67,68]. *E. coli* and *S. aureus* liquid cultures (50 mL) at the exponential growth phase (OD_600_ = 0.5, corresponding to approximately 4 × 10^8^ cells/mL) were centrifuged (20 min, 2.500 g, 4 °C), washed twice with sterile Phosphate Buffered Saline (PBS) and resuspended in an equal volume of PBS (50 mL). To ensure desired microorganism concentration (initial concentration of 10^6^ cells/mL) in the subsequent photocatalytic reactions, 25 μL of the bacterial suspension was used per 10 mL photocatalysis reactions. Volumes required for 10^6^ cfu/mL or pfu/mL of *B. stearothermophilus* spores and MS2, respectively, were calculated based on their previously determined titters.

Heterogeneous photocatalysis was performed in PBS, using 0.5 g/L TiO_2_. Homogeneous photocatalysis was performed in H_2_O pH 3, using 7 mg/L FeCl_3_ and 100 mg/L H_2_O_2_.

At different time points (0, 2, 5, 15, 30, 60, 90, 120, 180, 300 min, depending on the microorganism), samples (10–1000 μL) were collected and kept on ice and in dark until plated on appropriate nutrient media using the double layer agar method to determine microorganism inactivation. Specifically, for *B. stearothermophilus* spores, samples were boiled for 5 min to activate endospore germination, then mixed with 3 mL TSB top agar equilibrated at 60 °C and overlayed on TSB agar plates. Plates were incubated o/n at 60 °C, and the formed colonies were counted. For MS2, 10 μL from each sample were mixed by pipetting with 50 μL of a mid-log phase culture (OD_600_ = 0.5 − 0.8) of the *E. coli* Top10F’ host and incubated for 5 min at room temperature to allow bacterial infection. Then, samples were mixed with 3 mL LB-top agar equilibrated at 42 °C and overlayed on LB agar plates. Plates were incubated at 37 °C; the viable phage content (pfu) was quantified by counting the plaques formed on the bacteria lawn. For *S. aureus* and *E. coli*, collected samples were mixed with 3 mL LB top agar equilibrated at 42 °C, vortexed and spread on agar plates of the appropriate nutrient media (LB and LB-tet, respectively). Plates were incubated o/n at 37 °C, and the formed colonies were counted.

Negative control experiments were performed in the absence of a catalyst or in the presence of a catalyst, but without illumination. Each experimental procedure was performed in triplicates. For heterogeneous photocatalysis optimization, different TiO_2_ concentrations were tested; 0.1 g/L, 0.5 g/L and 1 g/L. The best-performing TiO_2_ concentration was then used in experiments supplemented with increasing concentrations of H_2_O_2_ (100 mg/L, 500 mg/L, 1000 mg/L).

### 4.4. Estimation of Photocatalytic Inactivation Efficiency

Microorganism viability reduction in relation to processing time was measured based on the ratio Nt/N0; Nt corresponds to the cfu/mL or pfu/mL determined through colony or plaque counting for each microorganism at the tested time point; N0 corresponds to the initial cfu/mL or pfu/mL (time point 0). We plotted Nt/N0 ratios relative to the processing time per microorganism and condition for each sample and corresponding controls. In addition, we estimated the inactivation efficiency (I) for each sample and time point, based on the colonies or plaques (cfu/mL or pfu/mL) detected in the tested sample (s), relative to zero time (N_ts_/N_0s_) and relative to corresponding controls I (N_tc_/N_0c_). The ratio $\frac{\mathrm{Nts}/N0s}{\mathrm{Ntc}/N0c}$ was used. For easier graphical representation the ratio $\frac{\mathrm{Nts}/N0s}{\mathrm{Ntc}/N0c}$ was multiplied by 10^5^ and expressed as log10 values; I $=\log\left(10^{5}\times \frac{\mathrm{Nts}/N0s}{\mathrm{Ntc}/N0c}\right)$.

## Figures and Tables

**Table 1:** Heterogeneous photocatalysis optimization.

$ Microorganism ^1^	TiO_2_ 0.1 g/L	TiO_2_ 0.5 g/L	TiO_2_ 1 g/L	TiO_2_ + H_2_O_2_ 100 mg/L	TiO_2_ + H_2_O_2_ 500 mg/L	TiO_2_ + H_2_O_2_ 1000 mg/L
*Bacillus stearothermophilus* spores	**180 min (3.1 ± 0.7)**	300 min (3.0 ± 0.6)	300 min (2.6 ± 0.5)	180 min (2.7 ± 0.3)	60 min (3.8 ± 0.1)	**60 min (5.0 ± 0.1)**
*Staphylococcus aureus*	**30 min (5.0 ± 0.2)**	**30 min ^2^ (4.8 ± 0.3)** **30 min ^2^ (4.8 ± 0.3)**	60 min (4.6 ± 0.3)	15 min (5.1 ± 0.0)	**5 min (5.0 ± 0.0)**	**5 min (5.1 ± 0.1)**
MS2	**15 min (4.4 ± 1.1)**	**15 min ^2^ (4.4 ± 1.1)**	15 min (2.7 ± 0.1)	**5 min (5.0 ± 0.0)**	**5 min (5.0 ± 0.0)**	**5 min (5.0 ± 0.0)**
*Escherichia coli*	**30 min (5.0 ± 0.1)**	**5 min (5.0 ± 0.1)**	**15 min (5.0 ± 0.1)**	**2 min (5.0 ± 0.0)**	**2 min (5.0 ± 0.0)**	**2 min (5.0 ± 0.0)**

^1^ Initial concentration: 10^6^ cfu/mL (*Bacillus stearothermophilus* spores, *Staphylococcus aureus*, *Escherichia coli*) or 10^6^ pfu/mL (MS2). ^2^ TiO_2_ concentration used for H_2_O_2_ testing. $ The table summarizes processing time and microorganism inactivation efficiency (I $=\log\left(10^{5}\times \frac{\mathrm{Nts}/N0s}{\mathrm{Ntc}/N0c}\right)$), mean and standard error from triplicates) under different optimization conditions. Different catalyst concentrations (0.1, 0.5, 1 g/L) were tested; the best performing catalyst concentration was further tested in the presence of H_2_O_2_ (100 mg/L, 500 mg/L, 1000 mg/L). Higher H_2_O_2_ concentrations significantly enhanced the inactivation efficiency of *B. stearothermophilus spores* and *S. aureus*; in the case of the more susceptible MS2 and *E. coli*, all tested H_2_O_2_ concentrations yielded similar results. The best performing conditions in each case are shown in bold.

## Data Availability

Not applicable.

## Supplementary Materials

The following supporting information can be downloaded at: https://www.mdpi.com/article/10.3390/molecules28031199/s1, Figure S1: Homogeneous and heterogeneous photocatalytic inactivation of tested microorganisms; Figure S2: Inactivation efficiency of homogeneous and optimized heterogeneous photocatalysis.
