# Supplementary material for: Photo-Fenton and TiO2 Photocatalytic Inactivation of Model Microorganisms under UV-A; Comparative Efficacy and Optimization
Source: Molecules. 2023 Jan 26;28(3):1199. doi: 10.3390/molecules28031199 (PMC9920570; doi:10.3390/molecules28031199)
Supplement: Supplementary file 1 [file molecules-28-01199-s001.zip › molecules-2120150-supplementary.pdf]

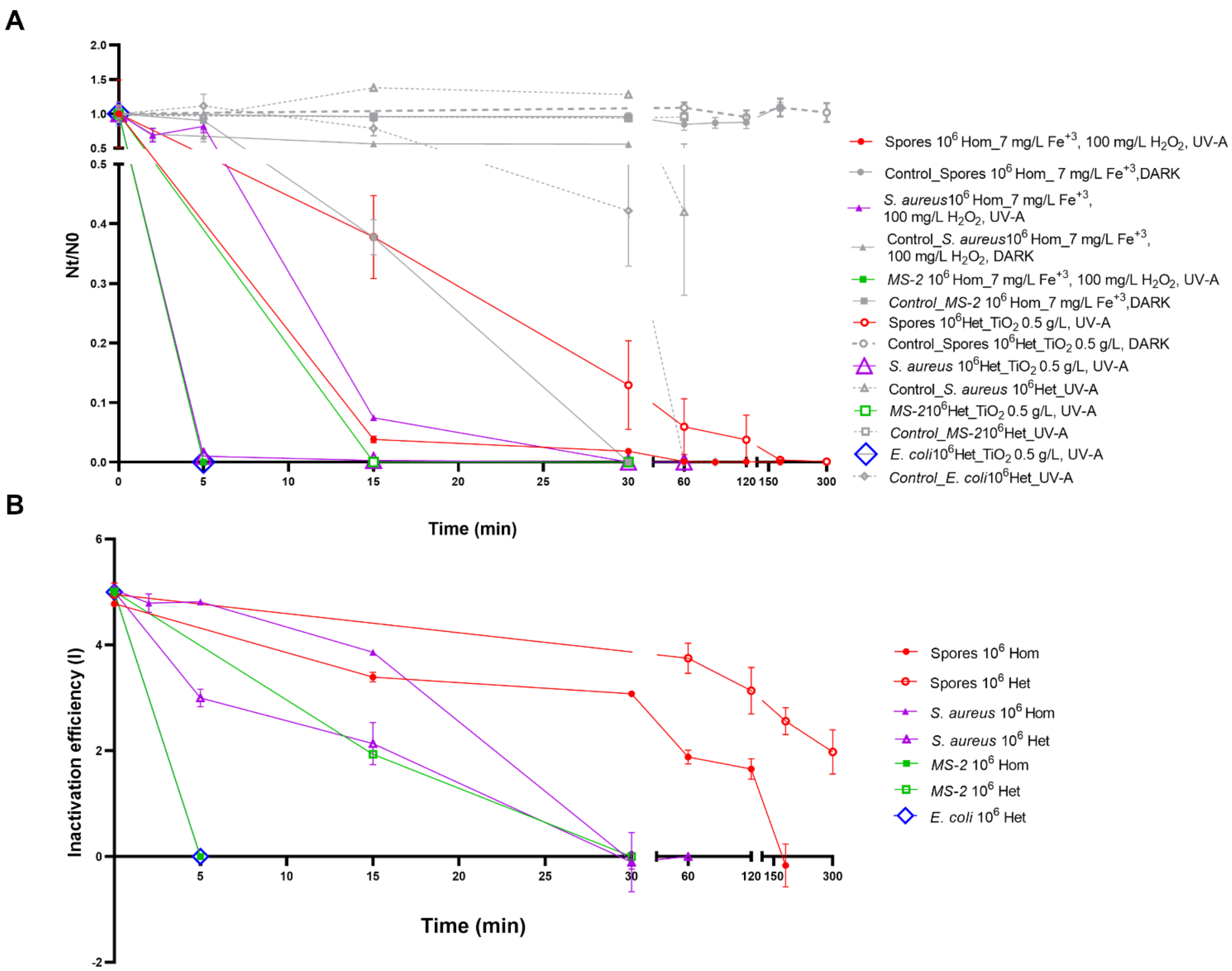

Figure S1: A. Homogeneous (7 mg/L Fe^{3+}, 100 mg/L H_2O_2, pH 3.0, UVA, solid symbols) and heterogeneous (0.5 g/L TiO_2 P25, UV-A, no fill symbols) photocatalytic inactivation of 10^6 cfu/ml *Bacillus stearothermophilus* spores (red), 10^6 pfu/ml *MS-2* (green), 10^6 cfu/ml *Staphylococcus aureus* (purple) and 10^6 cfu/ml *Escherichia coli* (blue). The graph depicts viability reduction (Nt/N0) in relation to processing time for each treated sample and corresponding control (grey lines). Zero values in the Y axis represent cases for which no microorganism growth was detected (0 cfu/ml or pfu/ml). The legend shows experimental conditions for processed samples and corresponding controls. *Escherichia coli* is highly susceptible at pH 3 required for homogeneous photocatalysis and thus not included in homogeneous photocatalysis experiments. **B.** Same data as in A, depicting inactivation efficiency (I) per sample and time point relative to corresponding controls. Error bars represent standard errors from triplicates. Homogeneous outperforms heterogeneous photocatalysis in terms of processing time required to achieve similar inactivation rates for all tested microorganisms, except from *Staphylococcus aureus*, where the same inactivation rate was achieved following 30 minutes processing in both cases.

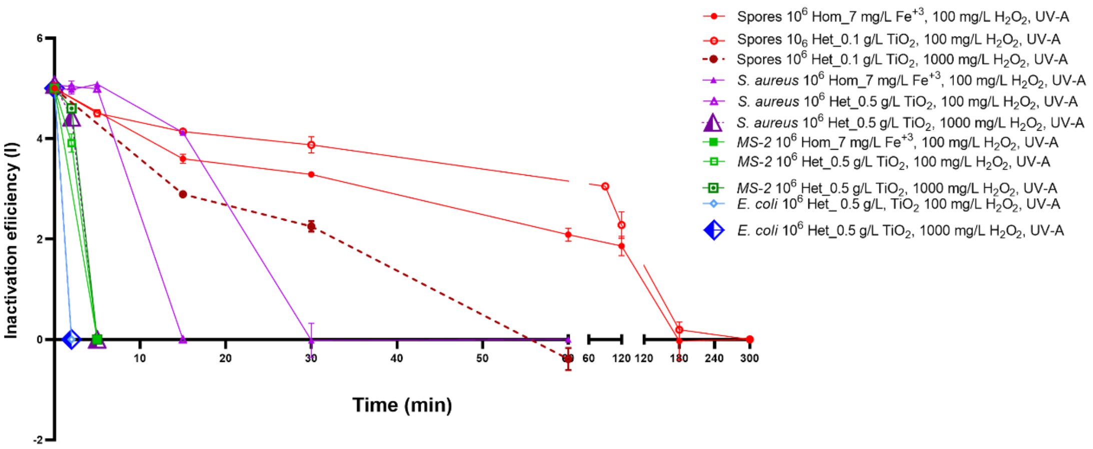

Figure S2: Homogeneous (7 mg/L Fe^{3+}, 100 mg/L H_2O_2, pH 3.0, UVA, solid symbols) and microorganism-dependent TiO_2 concentration optimized heterogeneous photocatalysis (no fill symbols) supplemented with either 100 mg/L H_2O_2 (continuous lines) or 1000 mg/L H_2O_2 (dashed lines) for the inactivation of 10^6 cfu/ml *Bacillus stearothermophilus* spores (red), 10^6 pfu/ml *MS-2* (green), 10^6 cfu/ml *Staphylococcus aureus* (purple) and 10^6 cfu/ml *Escherichia coli* (blue). The graph depicts Inactivation efficiency (I) in relation to processing time and corresponding controls. Zero values in the Y axis represent cases for which no microorganism growth was detected (0 cfu/ml or pfu/ml). Error bars correspond to standard errors from triplicates. The legend shows experimental conditions for processed samples. *Escherichia coli* is highly susceptible at pH 3 required for homogeneous photocatalysis and thus not included in homogeneous photocatalysis experiments.
